# Antimitotic drugs in the treatment of cancer

**DOI:** 10.1007/s00280-015-2903-8

**Published:** 2015-11-12

**Authors:** Rustelle Janse van Vuuren, Michelle H. Visagie, Anne E. Theron, Annie M. Joubert

**Affiliations:** Department of Physiology, University of Pretoria, Private Bag x 323, Arcadia, 0007 South Africa

**Keywords:** Taxanes, Epothilones, Vinca alkaloids, Estrogens, 2-Methoxyestradiol

## Abstract

Cancer is a complex disease since it is adaptive in such a way that it can promote proliferation and invasion by means of an overactive cell cycle and in turn cellular division which is targeted by antimitotic drugs that are highly validated chemotherapy agents. However, antimitotic drug cytotoxicity to non-tumorigenic cells and multiple cancer resistance developed in response to drugs such as taxanes and vinca alkaloids are obstacles faced in both the clinical and basic research field to date. In this review, the classes of antimitotic compounds, their mechanisms of action and cancer cell resistance to chemotherapy and other limitations of current antimitotic compounds are highlighted, as well as the potential of novel 17-β estradiol analogs as cancer treatment.

## Introduction

For 2015, 1.658 million new cancer cases and 589,430 deaths were predicted worldwide and, according to the National Cancer Registry (NCR), more than 100,000 South Africans are annually diagnosed with cancer with a survival rate of 60 % [1, 2].

**Fig. 1:**
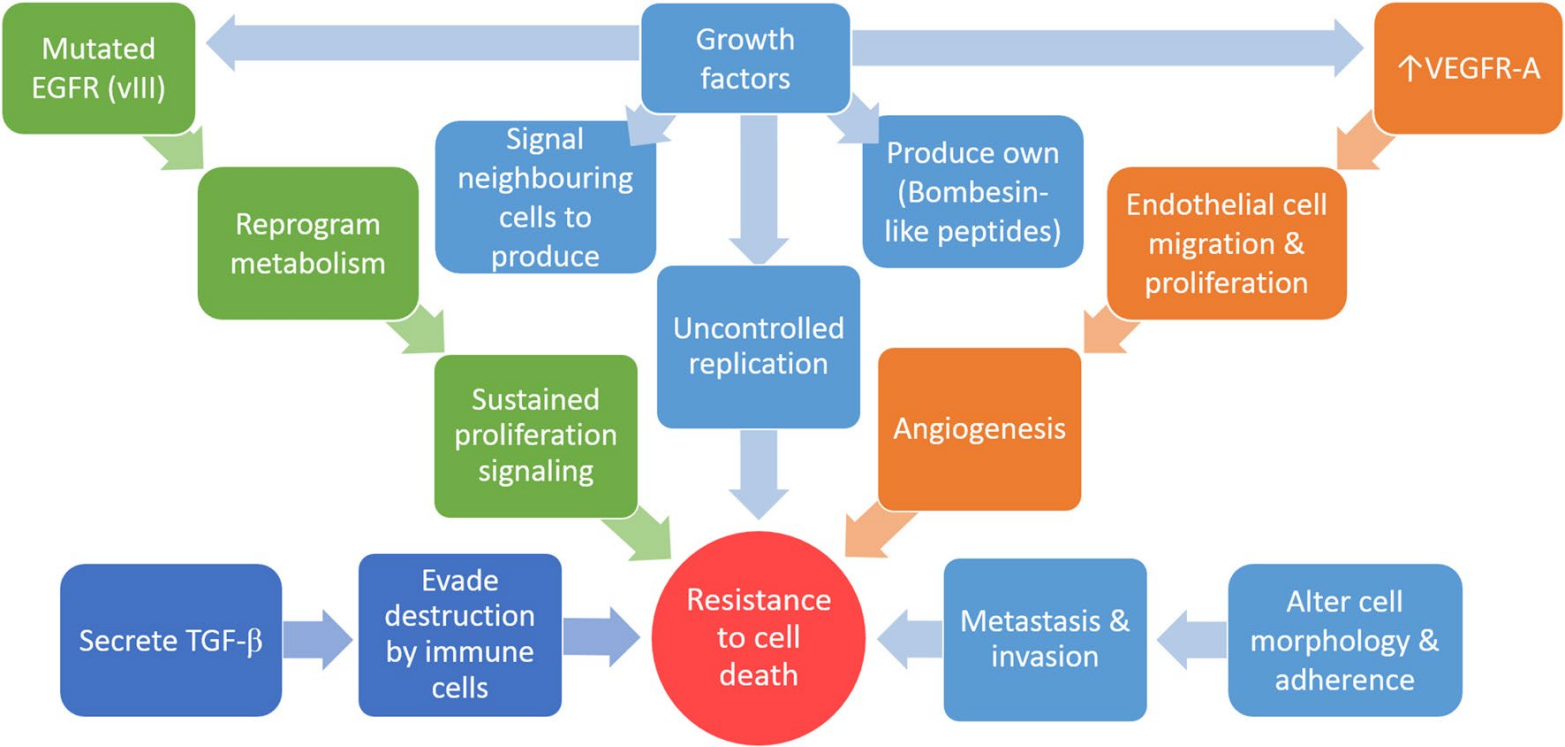
Cancer cells have the ability to evade anti-proliferating signals sent from surrounding tissues, sustain proliferative signals and avoid cell death which enable continuous replication, active metastasis and invasion and induce angiogenesis. Images were created using Microsoft^®^ PowerPoint^®^ 2013 software Pty/Ltd

Cancer refers to abnormal growth or malignant tumors and is characterized by uncontrolled proliferation of cells despite restriction of nutrients and space [3]. Cancer cells have unlimited replicative potential via the upregulation of telomerase (a specialized deoxyribonucleic acid (DNA) polymerase) expression that counters telomerase erosion (Fig. 1) [4].

In addition, cancer cells have the ability to evade tumor suppressor genes, resulting in sustained chronic proliferation. These cells may avoid apoptosis induction by the loss of protein p53 (TP53) tumor suppressor function, or in the case of necrosis, proinflammatory signals that recruit cells of the immune system, which may promote malignancy and invasion [3, 5]. Cancer cells may also produce their own growth factor ligands such as Bombesin-like peptides (secreted by human small cell lung cancer) or signal to non-tumorigenic surrounding tissue to supply cancer cells with growth factors (Fig. 1) [6]. These cells may activate invasion and metastasis by developing alterations in shape and attachment to the extracellular matrix and neighboring cells (Fig. 1) [3].

Tumorigenic cells can induce angiogenesis by upregulation of vascular endothelial growth factors, such as vascular endothelial growth factor A (VEGF-A) by either hypoxia or oncogene signaling which stimulates endothelial cell migration and proliferation (Fig. 1) [7]. VEGF along with other factors recruit tumor-associated macrophages and other factors including chemokine (C–C motif) ligand 2 (CCl2) chemokine (C–C motif), ligand 5 (CCL5), colony-stimulating factor 1 (CSF-1), endothelins (ET-1) and transforming growth factor beta (TGF-β) which stimulate cancer cell proliferation, invasion and angiogenesis [7].

The mutated form of endothelial growth factor receptor (EGFRvIII) supports chronic proliferation by enabling cells to reprogram their cellular metabolism to keep up with high energy demands [8]. In virus-induced cancers and some non-viral etiology cancers, cells have the ability to evade destruction by immune cells, especially, T- and B-lymphocytes, natural killer cells and macrophages [9, 10]. These cancer cells may secrete immunosuppressive factors such as TGF-β, or block interferon gene transcription or their promoters [3]. In addition, tumor cells recruit cells that are actively immunosuppressive, such as regulatory T cells, or suppress capsid protein production and subsequently immune cell detection [9]. Current cancer treatment includes an array of treatment options and regimens that are specific for each cancer type. Treatment efficacy has inter-individual variability which will be discussed below.

### Overview of current treatment

Current cancer treatments that are quite common include chemotherapy, radiation and surgery. Another less established treatment is immunotherapy, where biotherapy results in the increased recognizability of cancer cells by immune cells [11]. Immunotherapy includes cancer vaccines (either prophylactic or therapeutic vaccines) that reprogram memory T cells and increase cancer autologous (Ag)-specific effector T cells in vivo [12]. Targeted therapies are specifically aimed at cancer-associated molecules. These include rituximab (Rituxan^®^) and ibritumomab (Zevalin^®^) that target anti-CD20 antibodies on non-Hodgkins lymphoma cells [13].

Antimitotic drugs inhibit polymerization dynamics of microtubules (paclitaxel and vinblastine) by activating the spindle assembly checkpoint (SAC) blocking transition from metaphase to anaphase [14]. Subsequently, cells undergo mitotic arrest and since the compound disrupts spindle formation and chromosome orientation, cells remain either in a prolonged arrest state with subsequent apoptosis induction or in a senescence-like G_1_ state [15]. Microtubules are formed during interphase and are vital for correct chromosome segregation and cell division undergoing mitosis [16]. Microtubule dynamics is faster during mitosis compared to interphase, and thus microtubules are an ideal drug target since cancer cells possess hyperproliferative activity [16].

### Mechanism of action of antimitotic drugs

Drugs that act on microtubules can be divided into two groups according to their mechanism of action as either microtubule-destabilizing agents or microtubule-stabilizing agents [17]. Destabilizing drugs inhibit the polymerization of microtubules when administered at high concentration [18]. Most destabilizing drugs bind to either the vinca domain or taxoid-binding domain [16]. Those that bind to the vinca domain found in the interface between β- and α-tubulin (called vinca alkaloids) include vinflunine, vincristine, vinorelbine, vindesine and eribulin [19, 20]. Those that bind to the colchicine domain include cryptophycins, dolastatins and combretastatin-A4 [21, 22]. Drugs that enhance microtubule polymerization when administered at high concentrations, stabilize microtubules and prevent Ca^2+^- or cold-induced depolymerization, and subsequent disassembly, include eribulin, spongistatin, rhizoxin, maytansinoids second- and third-generation taxanes, epothilones, ixabepilone and many others [16, 23]. Taxanes, epothilones and many others belonging to this group bind to the inner surface of the microtubule at a taxoid-binding site on β-tubulin [16, 23]. Compounds that bind to an overlapping non-vinca and non-taxoid site on drug-resistant βII- and βIII-tubulin isotypes include the microtubule-stabilizing drugs; peloruside A (PLA) and laulimalide (undergoing pre-clinical study) [24]. PLA and laulimalide binding results in a mitotic arrest at the G2/M phase of the cell cycle and subsequently cell death [24]. Another characteristic that makes these compounds superior to taxanes and vinca alkaloids is that they are poor substrates of P-gp drug efflux pumps [25, 26].

### Cell cycle targets

**Fig. 2:**
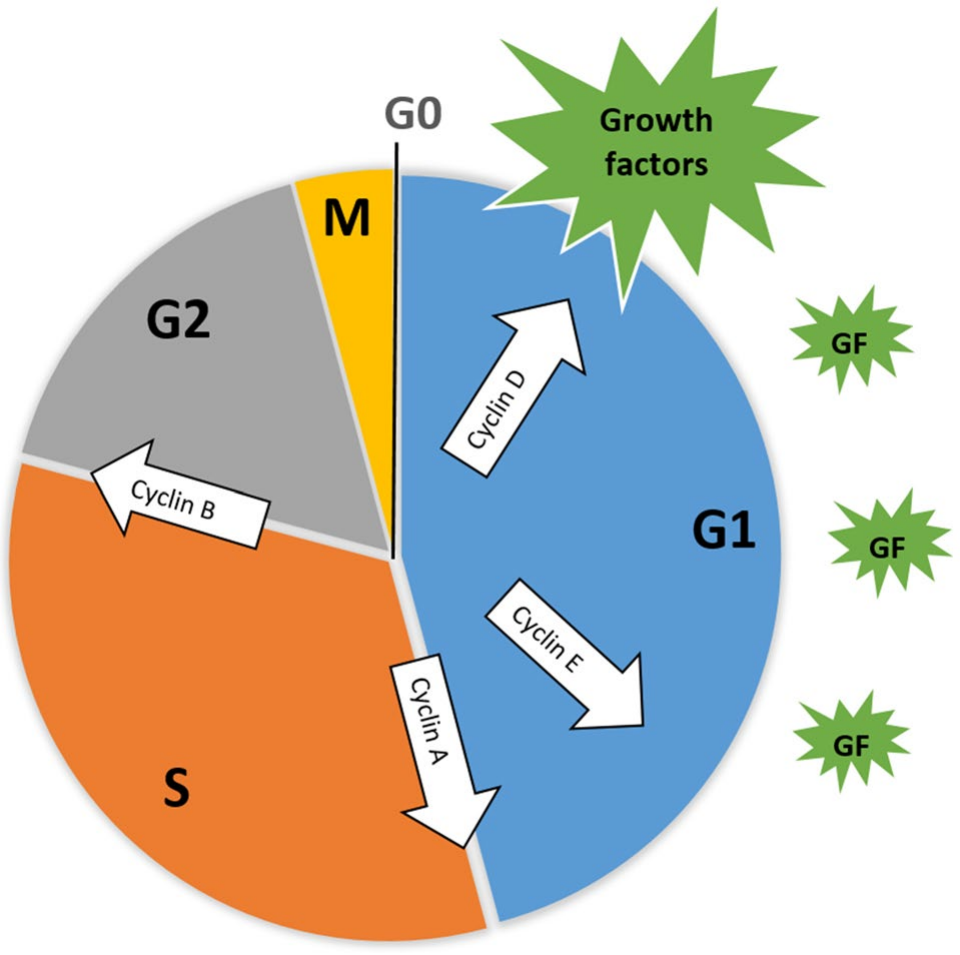
Cell cycle control by the expression of growth factors (*green*), primarily in the G_1_ phase. Internal cell cycle signaling regulates the expression of different cyclin proteins (*white arrows*) at different stages of the cycle. Images were created using Microsoft^®^ PowerPoint^®^ 2013 software Pty/Ltd

Cell cycle control is maintained by cyclin-dependent protein kinases (Cdks) which are activated when binding to cyclin proteins [27]. Different cyclin proteins are expressed at different stages of the cell cycle and form cyclin-Cdk complexes that initiate growth, mitosis and cytokinesis depending on the cyclin being expressed (Fig. 2) [28].

Cyclin/Cdk activity is regulated by factors including the DNA-binding transcription factors elongation factor 2 1-8 (E2F 1-8) and pocket proteins produced by the retinoblastoma tumor suppressor gene (pRB) responsible for the synthesis of cyclin proteins, cyclin-dependent kinase inhibitors (Cdki), phosphorylation status, proteolysis via ubiquitylation and subcellular localization in the nucleus or cytosol [28]. Cyclin D transcription is activated by growth factors and combines with cyclin-dependent protein kinase 4 (CDK4) [28]. The activation of Cdk4, when in complex with cyclin D, activates the E2F transcription system that aids in the induction of events resulting in DNA synthesis at the interface of the G_1_ and S phase [29]. After DNA replication (S phase), the cell enters another growth phase, G_2_, and activation of the cyclin B/Cdk1 complex induces entry into mitosis [28]. Two major interfaces exist within the cell cycle, namely the G_1_/S- and G_2_/M phase. Metaphase-to-anaphase interface is ensured by checkpoints including cell dimension and nutrient availability, DNA replication, DNA damage and spindle attachment [28].

### Spindle assembly

Accurate chromosome segregation during mitosis is ensured by feedback control via the spindle assembly checkpoint [31]. Correct spindle formation occurs when the sister kinetochores are connected to microtubules from opposite poles resulting in a bi-oriented chromosome or amphitelic attachment [31]. Incorrect chromosome segregation may result in aneuploidy and chromosome instability which is a characteristic of many aggressively proliferating tumors [32].

When a spindle fiber attaches to the kinetochore on a chromatid, the mitotic checkpoint complex (MCC) senses the tension between connected kinetochores and spindle fibers, as well as the lack of tension across unattached kinetochores and non-amphitelic attachments [33]. Unattached kinetochores signal MCC to inhibit anaphase-promoting complex/cyclosome (APC/C) [34]. The unattached kinetochore is then activated by Aurora kinase B and the active kinetochore recruits mitotic arrest deficient 1 (Mad1), budding uninhibited by benzimidazole (bub1) and multipolar spindle 1 (MPS1) [34, 35]. Aurora kinase B also modulates the Rod-Zwilch Zw10 (RZZ) complex which is involved in the recruitment process of Mad1 [31]. Mad1 binds to the unattached kinetochore and recruits mitotic arrest deficient 2 (Mad2) in closed formation resulting in the formation of more Mad2 proteins in a closed formation from Mad2 proteins in an open conformation [36]. The Mad2 proteins (closed formation) form a complex with mitotic checkpoint serine/threonine protein kinase Bub1 beta (BubR1), mitotic arrest deficient 3 (Mad3) and budding uninhibited by benzimidazole 3 (Bub3) resulting in cell-division cycle protein 20 (Cdc20) inhibition via phosphorylation and subsequently cannot bind to the anaphase-promoting complex cyclosome (APC/C) nor activate the mitotic proliferating factor (MPF) or degrade securing [37]. The cell enters mitotic arrest until proper spindle attachment has occurred at metaphase, and dynein is activated [27, 30]. Dynein is a motor protein that removes the MCC complex from the attached kinetochore [38]. Cdc20 is thus no longer inhibited, and active cdc20 is ubiquitinated by APC [27, 30]. Cdc20 activation of APC/C degrades securin (a protein responsible for the inhibition of the protein separase) via ubiquitination [39]. Separase cleaves and deactivates cohesion allowing sister chromatids to dissociate from one another and the cell enters anaphase [40].

Antimitotic drugs activate the spindle assembly checkpoint (SAC), since they disrupt microtubule formation and chromosome segregation resulting in the characteristic mitotic arrest [15]. Since the compounds are disruptive to the correct attachment of microtubules, the cells undergo cell death via apoptosis [15].

### Apoptosis

Apoptosis (adenosine triphosphate-dependent form of cell death) may occur through four different pathways, namely the intrinsic-, extrinsic-, endoplasmic reticulum-induced and the perforin/granzyme pathway [40].

The intrinsic pathway is usually governed by the B-cell lymphoma protein 2 (Bcl-2) protein family that can either be pro- or anti-apoptotic [41]. Pro-apoptotic proteins of the Bcl-2 family include Bcl-2-associated x protein (Bax), BH3 interacting domain death agonist (bid), Bcl-2 antagonist of cell death (Bad), Bcl interacting protein (Bim), Bcl-2 interacting killer (Bik), Bik-like killer protein (Blk) and snf B-cell lymphoma protein 10 (Bcl10) [41]. Bcl-2 proteins are responsible for mitochondrial membrane disruption and are regulated by tumor suppressor p53 [42]. Pores form in the mitochondrial membrane resulting in the reduction of the electrochemical gradient across the membrane [43]. The water-soluble heme protein, cytochrome complex (Cyt *c*) and serine protease Htr A2/Omi are transported from within the mitochondria through the disrupted outer membrane into the cytosol increasing effector caspases activity [44]. Cyt *c* binds apoptotic protease activating factor (Apaf-1) and cysteinyl aspartic acid-protease 9 (procaspase 9), thereby activating procaspase 9 [41].

In human cancer, defects in the control of apoptosis that lead to the protection of cancer cells to apoptotic stimuli are critical in tumor development [45]. Overexpression of anti-apoptotic or pro-survival proteins of the Bcl family such as Bcl 2, B-cell lymphoma-extra large (Bcl-x_L_), myeloid cell leukemia 1 (Mcl-1), Bcl-2-like protein 2 (BCL2L2 or Bcl-w) and Bcl-2-related protein A1 (A1/Bfl-1) has been reported to be present in cancer [45]. Overexpression of each of these above-mentioned proteins is associated with different tumor types, for example Bcl-x_L_ in multiple myeloma and Bcl-w in gastric cancer cells [46, 47]. Bcl-2 overexpression occurs in 90 % of colorectal cancer, 80 % of B-cell lymphomas, 70 % of breast and 30–60 % of prostate cancer [48]. The tubulysin analog, KEMTUB10, binds tubulin at the vinca domain inhibiting tubulin polymerization [49]. KEMTUB10 triggers apoptosis in MCF-7- and MDA-MB-231 cells by p53 upregulation and downregulation of Bim [49]. Bcl-2 overexpression confers cancer cell resistance pertaining to taxanes and, since KEMTUB10 does not prominently rely on Blc-2 phosphorylation to induce apoptosis, the compound is less susceptible to acquired Bcl-2 resistance [49].

The extrinsic pathway involves transmembrane receptors that form part of the tumor necrosis factor (TNF) receptor gene superfamily called death receptors [41]. Death receptors and their corresponding ligands are fatty acid synthetase receptor (FasR) and fatty acid synthase ligand (FasL), tumor necrosis factor receptor 1 (TNF R1) and tumor necrosis factor alpha (TNF-α), death receptor (DR) 3 and Apo3 ligand (Apo3L), DR4 and Apo2L, and DR5 and Apo2L [50]. When these receptor-ligand complexes form, cytoplasmic adaptor proteins are recruited, including Fas-associated death domain (FADD) in the case of the FasL-RasR complex and TNF receptor-associated death domain (TRADD) in the case of the TNF-α-TNFR1 complex [51, 52]. The latter results in death-inducing signaling complex (DISC) formation, subsequent activation of caspase 8 and the induction of the execution pathway [53]. The execution pathway is induced by the activation of executioner caspase 3 and subsequent DNA degradation, chromatin condensation, cell shrinkage, apoptotic body formation and membrane blebbing [41].

The taxane taxol induces the extrinsic pathway by upregulating Aurora-A (Aur-A) which phosphorylates FADD at S203 and subsequently induces DISC formation in human cervical adenocarcinoma cell line (Hela), human gastric adenocarcinoma cell line (AGS) and human colorectal adenocarcinoma cell line (HTC15) [54]. Aur-A phosphorylation of FADD at S203 allows for FADD S203A phosphorylation by polo-like kinase 1 (Plk1) [54]. The double-phosphorylated FADD (FADD-DD) also dissociates from, and subsequently activates, receptor-interacting serine/threonine protein (RIP1) inducing the caspase-independent apoptotic pathway [54]. Several above-mentioned proteins including Bcl-2 and p53 are involved in another cell death and survival pathways, namely autophagy that will be discussed below.

### Autophagy

Autophagy is a form of cell death where organelles and proteins are degraded resulting in energy that is packaged into double membrane vesicles known as autophagosomes [56]. Autophagic vesicles are transported along microtubule tracks fusing with lysosomes for degradation and recycling [55]. Autophagic pathways are upregulated when non-tumorigenic cells have a higher energy demand, such as nutrient deprivation, resulting in a stress state [55]. Cancer cells are resistant to autophagy by shrinking and entering a reversible dormant state when highly stressed due to the upregulation of autophagy by stressors such as starvation and chemotherapeutic drugs [55]. Through this mechanism, autophagy has been shown to support the survival of late stage or established tumors [3, 55].

Autophagic vesicles are transported by means of microtubules. Antimitotic drugs, disrupting the microtubule formation, result in vesicle accumulation, since they inhibit their fusion with lysosomes and thus their degradation and substrate recycling [55].

The taxane paclitaxel has been reported to inhibit autophagy in MCF-7 (a tumorigenic estrogen receptor-positive (+) cell line) and SK-BR-3 breast cancer cells that have entered mitosis by blocking the class III phosphatidylinositol 3-kinase vacuolar protein sorting protein 34 (Vps34), a protein vital in induction of autophagosome formation [55]. In MCF-7 and SK-BR-3 cells that were not undergoing mitosis because of mitotic slippage, paclitaxel prevented autophagy by hindering autophagosome trafficking [55].

## Classes of antimitotic drugs

### Taxanes

Taxanes are commonly used as chemotherapy treatment for breast cancer [57]. The taxane paclitaxel (taxol^®^) used in combination with carboplatin (an alkylating agent that has cytotoxic activity) is a common treatment regimen for lung carcinoma (Table 1) [58]. Paclitaxel inhibits microtubule depolymerization by binding to β-tubulin, resulting in mitotic arrest and subsequent activation of caspase-dependent apoptosis by Bcl-2 proteins [56]. Taxanes usually increase the patients’ survival in carcinoma of the lung, breast and ovaria. However, taxanes are also associated with side effects, namely peripheral neuropathy, myelosuppression, arthralgias and skin reactions including flushes and rashes (urticarial) [58, 59]. Since these side effects accumulate throughout the course of therapy and affect the patient’s quality of life, adjunctive medications are required to minimize subsequent side effects [57].

**Table 1:** Classes of antimitotic drugs and their stages of development [25, 26, 58, 61, 65, 67, 70, 71, 83, 85, 105–107]

Class	Name	Mechanism of action	Approved for treatment of (cancer type)
Drugs used as cancer treatment regimens			
Taxanes	Paclitaxel (taxol^®^)	Microtubule-stabilizing	Metastatic adenocarcinoma of the pancreas (in combination with gemcitabine)
	Cabazitaxel (Jextana^®^)	Microtubule-stabilizing	Metastatic, hormone-resistant prostate cancer (in combination with prednisone)
Epothilones	Ixabepilone (Ixempra^®^)	Microtubule-stabilizing	Metastatic or locally advanced breast cancer (resistant to taxanes and anthracycline)
Vinca alkaloids	Eribulin (E7389, ER086526, 6)	Microtubule-destabilizing	Recurrent metastatic breast cancer (pre-treated with taxanes and anthracycline)

Class	Name	Mechanism of action	Phase of clinical trials
Drugs undergoing clinical trials			
Vinca alkaloids	Vintafolide (EC145)	Microtubule-destabilizing	In Clinical phase II trials as sole treatment for ovarian and lung cancer

Class	Name	Mechanism of action	Model
Drugs undergoing in vivo studies			
Non-taxoid site microtubule-stabilizing agents	Peloruside A (PLA, CHEBI:77692)	Microtubule-stabilizing	Lung and breast tumor xenograft studies in athymic nu/nu mice
	Laulimalide	Microtubule-stabilizing	High toxicity and low tumor inhibition in human breast cancer and fibrosarcoma xenograft studies in athymic NCr-nu/nu mice

Class	Name	Mechanism of action	Effective in cell line
Drugs undergoing in vitro studies			
Estrogen derivatives	ESE-15-ol	Microtubule-destabilizing	Breast cancer (MCF-7, MDA-MB-231) and lung cancer (A549)
	ESE-16	Microtubule-destabilizing	Breast cancer cell lines (MCF-7, MDA-MB-231) and esophageal cancer (SNO)

Efficacy of taxanes as adjuvant therapy in early breast cancer is unclear [57]. Data of one clinical trial suggest that an addition of paclitaxel to anthracycline (an antibiotic class of chemotherapy that is cell-cycle non-specific) was only beneficial for women who had an overexpression of the human epidermal growth factor receptor 2 (HER2) in tumors of early breast cancer [57]. HER2 signaling influences multiple forms of taxane resistance including cell survival, as well as drug efflux and drug metabolism [60].

Cabazitaxel (Jextana^®^), a new microtubule-stabilizing taxane has been effective against metastatic breast- and metastatic hormone-resistant prostate cancer that acquired resistance to both paclitaxel and docetaxel [61]. Cabazitaxel has been improved by decreasing multidrug-resistant protein recognition for the compound and in turn reducing potential cancer cell resistance [61]. The antimitotic drug was approved for the treatment of metastatic, hormone-resistant prostate cancer in Europe (March 2011) (Table 1) [62]. Side effects of cabazitaxel include nausea, diarrhea, vomiting and neurotoxicity [61].

### Epothilones

Epothilones A and B were initially found in mycobacterium *sorangrum cellulosum* as cytotoxic metabolites that stabilize microtubules. Epothilones show higher cytotoxicity than taxanes in vitro [63]. For example, epothilone B shows a higher cytotoxicity to human ovarian cancer cells (OV-90) when compared to paclitaxel [64]. Epothilone B competitively inhibits paclitaxel, since both bind at the same site on tubulin-β [64]. However, epothilones and taxanes show no common mechanisms of resistance [64]. Epothilones are effective in cancers overexpressing class III β-tubulin where taxane resistance is attributable to the overexpression of class III β-tubulin [64].

Ixabepilone (Ixempra^®^) is a lactam analog of epothilone B (Table 1) [63]. The compound was approved by the USA in 2007 for use in the treatment of metastatic or locally advanced breast cancer that is resistant to taxanes and anthracycline [65]. The agent was the first epothilone to be approved for clinical use. Ixabepilone is metabolically more stable than its precursor, epothilone B, and thus the most clinically advanced epothilone with regards to its efficacy and tolerability in breast cancer patients [63]. Ixabepilone cytotoxicity is decreased cell lines expressing P-glycoprotein (P-gp), namely Madin-Darby canine kidney cells transfected with the human multidrug resistance 1 gene (MDCK-MDR1) and pig kidney epithelial cells transfected with the human multidrug resistance 1 gene (LLCPK-MDR1) [66]. The latter thus confirms that ixabepilone is a substrate of the ATP-binding cassette efflux transporter, P-glycoprotein (P-gp/MDR1/ABC1) such as taxane class [66]. However, ixabepilone is not a substrate of the breast cancer resistance protein (BCRP1/ABCC-2), a protein that is significantly overexpressed in doxorubicin- and paclitaxel-resistant breast cancer cells (MCF-7/DOX and MCF-7AX), which explains the potency of ixabepilone- in taxane-resistant breast cancer [66].

### Vinca alkaloids

The first vinca alkaloids were extracted from the plant *catharamthus roseus*, native to Madagascar, and were found to possess anticancer activities in 1960 [20]. Vina alkaloids include vincristine which was approved as chemotherapy treatment in 1963 in the USA [20]. These compounds bind to β-tubulin close to the guanosine triphosphate (GTP)-binding sites (the vinca domain) at the β-α-tubulin heterodimers interface [20]. Binding at the vinca domain prevents curved tubulin from straightening and, in turn, interferes with growth and assembly of microtubules [67]. Eribulin (E7389, ER086526, 6), a compound derived from marine sponge, was approved in 2010 in the USA as the third-line treatment for patients with recurrent metastatic breast cancer (pre-treated with taxanes and anthracycline) (Table 1) [20, 67]. However, treatment was accompanied with neutropenia and fatigue, and the lower occurrence of peripheral neuropathology compared to older drugs is a potential benefit of eribulin [68]. Unfortunately, the drug is a substrate for the P-gp efflux pump and may demonstrate decreased activity against cancer cells that overexpressed these efflux pumps [69]. Vintafolide (EC145) has recently shown promise in ovarian and lung cancer during phase II clinical trials as sole treatment (Table 1) [70, 71]. The compound consists of the microtubule-destabilizing agent desacetylvinblastine hydrazide, folic acid, a hydrophilic peptide spacer and a disulfide-containing self-immolative linker [72]. Vintafolide delivers the microtubule-destabilizing agent to the folate receptors (FR) of FR-tumor cells [72]. FR is overexpressed in various carcinomas and mediates the uptake of folic acid-conjugated compounds via endocytosis [73]. Once vintafolide is taken up in the cell, the disulfide bond is cleaved and active desacetylvinblastine hydrazide diffuses through the endosome to the cytoplasm where it causes microtubule disruption [74]. Since FR is expressed in small amounts in non-tumorigenic tissues it is an ideal tumor target [73].

### Microtubule-targeting estrogen derivatives

Estrogen aids in the growth, differentiation and maintenance of many tissues in the body including breast, uterine, cardiovascular, brain and urogenital tract tissues of both sexes by activating the nuclear estrogen receptors (ER), ERα and ERβ, to induce transcription factor activation [75, 76]. In various types of cancer, especially breast and ovarian cancer, estrogen is known to promote proliferation and invasion [75]. The goal for using estrogen-derived anticancer compounds is to compete with estrogen for the binding to estrogen receptors (ER) with antagonistic activity [75]. Fulvestrant (ICI182780) is an example of such a compound and shows to be more effective when compared to tamoxifen, the current non-steroidal anti-estrogen compound used as standard hormone treatment for breast cancer [75, 77].

In postmenopausal woman, estrone sulfate is found in high concentrations in breast tissue (3.3 ± 1.9 pmol/g vs. premenopausal woman 1.2 ± 0.3 pmol/g) and more so in patients with breast carcinoma [76–79]. The concentration of estrone sulfate is up to seven times higher in breast tissue than in plasma and is converted to estradiol sulfate in hormone-dependent breast cancers via the 17-β-hydroxysteroid dehydrogenase type 1 enzyme [79, 80]. The majority of breast cancer begins as a hormone-dependent cancer where estradiol plays a vital role in tumor growth and development [81].

### 2-Methoxyestradiol and in silico-designed analogs

2-Methoxyestradiol (2ME), an analog of 17-β estradiol, occurs naturally in the human body and exerts antimitotic activity [44]. 2ME binds at the colchicine domain of β-tubulin in microtubules resulting in microtubule depolymerization [44, 82]. At low concentrations, 2ME destabilizes microtubules and impairs correct spindle-kinetochore attachment; the cell subsequently undergoes cell death as a result of prolonged mitotic arrest [82]. The 17-hydroxy group pertaining to 2ME makes it a target for 17-hydroxysteroid dehydrogenase-mediated metabolism (in the gastrointestinal tract (GIT) and liver) resulting in rapid metabolism and subsequent low bioavailability [83]. 2-Ethyl-3-*O*-sulfamoyl-estra-1,3,5(10)15-tetraen-17-ol (ESE-15-ol), 2-ethyl-3-*O*-sulfamoyl-estra-1,3,5(10)16-tetraene (ESE-16) and 2-ethyl-3-*O*-sulfamoyl-estra-1,3,5(10),15-tetraen-3-ol-17-one are sulfamoylated analogs of 2ME and have been in silico designed in order to selectively bind to and inhibit carbonic anhydrase IX (CAIX) in vitro [83, 85]. CA IX, a zinc membrane-bound enzyme, is upregulated in most types of cancer and acidifies the extracellular environment by converting carbon dioxide and water to carbonic acid [86]. Acidification of the cancerous environment promotes further metastasis and invasion [83]. The acidification of the extracellular environment may also lead to chemoresistance, since the uptake of weakly basic anticancer drugs is decreased by the formation of a H^+^ gradient across the cellular membrane [86].

CAIX is also involved in cellular migration and invasion of human cervical carcinoma cells (C33A) in vitro [87]. In non-tumorigenic physiological conditions, this metalloenzyme is only found in a few non-tumorigenic tissues such as coelomic epithelial cells, basal cells in and around hair follicles, gastric mucosa cells and cells in the ventricular lining of the choroid plexus [86, 88]. During carcinogenesis, the expression of CAIX in these tissues is either reduced or lost [86]. Since CAIX is predominantly expressed in carcinomas from non-tumorigenic tissues that do not express CAIX, it is an ideal protein marker for cancer [86]. The upregulation of CAIX is induced by hypoxia via the transcription factor, hypoxia-inducible factor-1 (HIF-1) [84]. 2ME inhibits HIF-1 target gene expression in tumor cells at the posttranscriptional level [88]. The alpha subunit of HIF (HIF-1α) is overexpressed in many human cancers [89]. 2ME blocks accumulation of HIF-1α in the nucleus and in turn prevents activation of several genes that are crucial for cell transformation and survival under hypoxic conditions [89].

Modifications to the chemical structures of these compounds, including the addition of a sulfamoylated group or the removal of a hydroxyl group, increase the bioavailability of these compounds as it prevents first pass metabolism by the liver [44, 85, 90]. In addition, this modification allows these compounds to bind to carbonic anhydrase II (CAII) in red blood cells, resulting in a slower release of these compounds into the bloodstream and in turn avoiding first pass metabolism [85]. These characteristics allow ESE-15-ol and ESE-16 to be potentially more effective than their rapidly metabolized precursor 2ME [83–85]. An increase in G_1_ phase (an indication of cell death via DNA damage), a decrease in mitochondrial membrane potential (an indication of apoptosis via the intrinsic pathway) and G_2_/M arrest, followed by disrupted spindle formation or the formation of multiple spindle poles, are events induced by ESE-15-ol and ESE-16 [83–85]. MDA-MB-231, a metastatic tumorigenic estrogen receptor-negative cell line, MCF-7 and MCF-12A, a non-tumorigenic estrogen receptor-negative (−) cell line, were used for evaluation. A general 50 % inhibition of cellular growth was seen across the three cell lines at nanomolar concentrations after 24 and 48 h exposure periods, proving the compounds are more potent than 2ME in vitro [83–85]. The compounds also had a reduced effect on the non-tumorigenic cell line, MCF-12A (−), when compared to the tumorigenic cell lines that are exposed to ESE-15-ol, and this was especially evident after 48 h [83–85].

In both ESE-15-ol and ESE-16 exposed cells, there was a disruption in phosphorylation of the pro-apoptotic binding protein, Bcl-2, at serine 70 in the MDA-MB-231 (−) cell line, indicating activation of apoptosis via the intrinsic pathway, corresponding with the decrease in mitochondrial membrane potential observed [83–85]. The studies thus demonstrated that these compounds possess potential as antimitotic agents with respect to potency and bioavailability in vitro (Table 1) [83–85].

### Cancer cell resistance to antimitotic compounds

Resistance to antimitotic drugs can occur at different stages of treatment, and the comprehension of these resistance mechanisms is vital in the development of novel antimitotic compounds [16]. Genetic changes that exist prior to treatment are the first cause of therapeutic failure of chemotherapy, and this is known as intrinsic or primary resistance [91]. Secondary or acquired resistance is a result of drug treatment [91].

One mechanism developed by tumor cells, when exposed to chemotherapy including antimitotic drugs in vitro, is the membrane efflux pumps of the ATP-binding cassette (ABC) family [16]. These transporters export the compounds that have accumulated within the cells through the cellular membrane avoiding toxicity of the drugs [16]. The multidrug resistance gene 1 (MDR1 or ABCD1) is responsible for the production of P-gp, part of the ABC family, which effluxes many hydrophobic antimitotic drugs such as taxanes and vinca alkaloids [16, 92]. ABCD1 and P-gp overexpression is involved in both intrinsic drug resistance and acquired drug resistance [91]. The multidrug-associated protein 1 (MRP1) transports vinca alkaloids out of the cell [16]. MRP2 and MRP7 are responsible for the export of taxanes and MRP7 for the transport of epothilone B [16]. Expression of these efflux pumps shows a correlation with a lower response to antimitotic chemotherapy in primary tumors [16]. Thus, developing drugs that are not substrates of P-gp, such as second- and third-generation taxanes and epothilones, whose structural modifications allow them to avoid P-gp, are essential to overcome the obstacles of cancer resistance [92]. Another strategy is to make use of molecules, where the activity is strengthened by overexpressed P-gp efflux pumps [91]. It has been reported that combination treatment of a multidrug-resistant breast cancer cell line (MCF-7/ADR) with paclitaxel and the P-gp inhibitor Verapamil had a synergistic effect on cytotoxicity in vitro [93].

Mutations in p53 gene expression, activating mutations of phosphatidyl-3-phosphate kinase (PI3 K) and gene expression of the Ras/Raf pathway all have been reported to result in increased resistance to antimitotic drugs in tumor cells [94]. Hypomethylation of phosphatase and tensin homolog deleted from chromosome 10 (PTEN), a tumor suppressor gene, destabilizes the gene and results in the upregulation of the phosphatidylinositol 3-kinase/Akt kinase (PI3 K/Akt) pathway, which activates Akt, a protein that regulates anti-apoptotic proteins and cell cycle entry resulting in survival signaling [91]. The loss of PI3 K regulation increases Bad phosphorylation, resulting in the deactivating of the pro-apoptotic protein and subsequently protects the mitochondrial membrane from disruption [94]. This increases resistance to cell death induced by the intrinsic pathway. The overexpression of the mitogen-activated protein kinase cascade (Ras/Raf/MAPK) pathway, where Ras (a small GTP kinase receptor) activates MAPK, results in the activation of Raf. Mutations of these genes that upregulate this pathway lead to survival signaling [91].

The overexpression of class III β-tubulin isotope, a marker used in the diagnosis of solid tumor malignancies such as ovarian and lung cancer, is suspected of being responsible for resistance to paclitaxel [95]. βIII-tubulin enhances microtubule dynamic instability and counteracts the stabilizing action of taxanes [17]. It also affects the efficacy of vinca alkaloids [91]. βIII-tubulin is expressed in stressed cells deprived of oxygen and nutrients [91]. The expression of βIII-tubulin is a survival pathway, that, when inhibited in nude mice, increases the sensitivity of cells to chemotherapy, but also inhibits colony formation and the development of tumorigenesis [17]. Mutations in the β-tubulin gene in vitro and in patients also seem to contribute to drug resistance, specifically antimitotic compounds [94]. Regulating proteins of microtubules such as mitotic centrosome-associated kinesin (MCAK), stathmin and tau are associated with antimitotic drug resistance [91]. Deregulation of proteins of the SAC via gene amplification such as the protein Aurora kinase via AURORA-A amplification also contributes to resistance in drugs that target microtubules [96].

HER2 signaling activates the transcription factor Y-box-binding protein-1 (YBX), and in turn increases survival, reduces induction of apoptosis and enhances drug efflux [97, 98]. A positive feedback loop exists between HER2 and YBX that promotes further cancer cell immortality [91]. Thus, HER2 overexpression results in increasingly aggressive tumors and HER2-amplified cancer types pose resistance to taxanes by regulating P-gp efflux pumps [60]. The latter is accomplished by means of survivin, which is crucial in spindle assembly formation, and cyclin-dependent kinase inhibitor 1A (P21CIP1) that inhibits cell cycle progression at G_1_ [60]. Augmentation of HER2 occurs in 20–25 % of breast cancer types, and HER2-targeted therapy (trastuzumab and lapatinib) has been reported to increase life expectancy by 50 %. Reoccurrence after treatment is a major obstacle faced in the clinic, and the mechanisms of resistance to these compounds have not yet been confirmed [99]. Another factor influencing resistance is hypoxia, commonly found in the center of solid tumors [100]. Hypoxia potentially reduces drug access and efficacy [100]. This oxygen-deprived state in tumors influences cell cycle control signaling pathways and angiogenesis and increases invasion and metastasis [100]. Hypoxia also inhibits the intrinsic pathway of apoptosis by reducing the Bax/Bcl-2 ratio [100]. Since an increase in resistance due to hypoxia in the presence of paclitaxel is reversed by increased cyclin B1 levels, hypoxia reduces the antimitotic activity of paclitaxel by downregulation of cyclin B1 [100].

The non-coding microRNAs is another gene expression regulator found both over- and under expressed in several types of cancer including breast, prostate, lung, gastric, colon, ovarian cancer and leukemia. MicroRNA confers cancer cell resistance to antimitotic drugs since it regulates various genes involved in the cell proliferation, differentiation and apoptosis [101]. miR-125b is overexpression in taxol-resistant breast cancer cells, 435TRP and metastatic breast cancer cells, MDA-MB-231. miR-125b targets Bcl-2 antagonist killer 1 (Bak1), a pro-apoptotic protein, and confers resistance to antimitotics such as paclitaxel [102]. MicroRNAs also target Bcl-2, Bax, Bcl-xL and caspase 3 and 7 expression leading to the disruption of apoptosis [101]. Other microRNAs, such as miR-27, regulates the multidrug resistance 1 gene (MDR1) increasing drug efflux transporters (P-gp) and, in turn, confers resistance to its substrates including taxanes and vinca alkaloids [101].

The theory that antimitotic drugs target and kill cancer cells, because of their high proliferation rate in vitro, is contradicted with the low doubling time of tumor cells, such as primary breast cancer (40–300 days) and metastatic breast cancer (30–90 days) [15, 103]. To date, the mechanisms of anticancer drugs have predominantly been evaluated in cancer cell lines in vitro and mouse models with deficient immunity [104]. These models restrict research from determining the influence of these drugs on actual human tumor physiology, since they lack a representation of the immune system and vasculature [104]. This may lead to several action mechanisms going undetected.

## Conclusion

Antimitotic drugs such as the taxane cabazitaxel (Jextana^®^) (accepted in 2011), and the vinca alkaloid vintafolide (EC145, phase II), show promise in taxane and anthracycline-resistant cancers [71]. However, the toxicity of these drugs, as well as acquired drug resistance, allows for the opportunity to develop agents with increased tolerability and specificity [58, 59]. Development of novel compounds that disrupt mitosis without interfering with microtubule dynamics in non-dividing or highly proliferating (such as neutrophils) non-tumorigenic cells is the main focus in new antimitotic drug research. The in silico-designed 2ME analogs show promise since they were designed to target CAIX in the tumorigenic environment, increasing the bioavailability which will be evaluated in vivo in the near future [83–85].

Studies investigating the pathways of cancer cell resistance to antimitotic drugs will result in subsequent identification of novel biomarkers for future chemotherapy possessing increased efficacy. However, the limited success of antimitotics in clinical trials is mainly due to antimitotic targeting mechanisms varying substantially between in vitro and in vivo models since the drug resistance is poorly understood. In addition, unraveling the role of mitotic machinery and identifying the determinants of drug resistance in different models will contribute to the embedded scientific knowledge regarding antimitotic efficacy and subsequently yield novel biochemical targets for improved chemotherapy.
